# Evaluation of a general practitioner-based rehabilitation follow-up consultation to promote patients’ use of medical rehabilitation aftercare: study protocol for a pragmatic cluster randomized crossover trial

**DOI:** 10.1186/s12913-026-14938-9

**Published:** 2026-06-12

**Authors:** Matthias Lukasczik, Jennifer Seeger, Roland Küffner, Heiner Vogel

**Affiliations:** Center of Mental Health, Rehabilitation Sciences Section, University Hospital Würzburg, Margarete-Höppel-Platz 1, 97080 Würzburg, Germany

**Keywords:** Rehabilitation, Aftercare, Follow-up care, General practitioner, Cluster-randomized trial, Patient-provider communication, Patient motivation

## Abstract

**Background:**

The aim of the study is to investigate whether a structured follow-up consultation with the general practitioner (GP) contributes to an increased use of rehabilitation aftercare by patients after inpatient medical rehabilitation. The focus of the follow-up consultation four to eight weeks after rehabilitation is on supporting patients in implementing aftercare recommendations of their rehabilitation clinic and discussing barriers that prevent them from making use of aftercare services. The study will also investigate whether the intervention can encourage GPs to engage more intensively with medical rehabilitation and thus promote its long-term effectiveness.

**Methods:**

The study will be conducted as a two-period cluster randomized crossover trial using a mixed-methods approach. Participants in the intervention group and the control group will receive the usual recommendations for rehabilitation aftercare during their rehabilitation. Participants in the intervention group will also receive an invitation to a follow-up consultation with their GP. The primary outcome is the self-reported utilization of rehabilitation aftercare four months post-rehabilitation. Self-reported work ability four months post-rehabilitation is assessed as a secondary outcome. For the follow-up consultation, GPs will be provided with a written consultation guide, which will be developed through interviews with GPs. A written patient communication aid will be developed for patients. During the development process, patients will be interviewed to evaluate its comprehensibility and usability. Patient questionnaire data will be collected at three measurement points (T1: end of rehabilitation; T2: after follow-up consultation (intervention group only; four to eight weeks post-rehabilitation); T3: four months post-rehabilitation). Questionnaire data from GPs will be collected via online questionnaires after each follow-up consultation and once toward the end of the data collection period.

**Discussion:**

The study aims to investigate the extent to which a brief and low-threshold GP-centered intervention can contribute to an increased utilization of rehabilitation aftercare and thereby improve the sustainability of the effects of medical rehabilitation.

**Trial registration:**

Deutsches Register Klinischer Studien (German Clinical Trials Register); date: November 5, 2025; ID: DRKS00038353.

**Supplementary Information:**

The online version contains supplementary material available at 10.1186/s12913-026-14938-9.

## Background

Aftercare and follow-up programs are essential to maintain the therapeutic outcomes achieved during medical rehabilitation (MR) in terms of physical or mental health, functional status, work ability or behavioral/lifestyle changes in the everyday lives of MR patients. They aim to maintain or stabilize treatment outcomes and changes in health behavior (e.g., physical activity, weight reduction, smoking cessation), and to support reintegration and participation (in terms of the International Classification of Functioning, Disability and Health, ICF) [1].

The various national health and rehabilitation systems organize aftercare differently. Compared to other countries, the MR system in Germany has several distinctive features that are also relevant for rehabilitation aftercare. MR is mainly carried out as a three-to-four-week inpatient program in specialized rehabilitation centers with an indication-specific treatment focus and multi-professional rehabilitation teams, and is funded by different providers [2]. The German statutory pension insurance (GPI; in German: Deutsche Rentenversicherung, DRV) provides MR for persons of working age. Its goal is to support successful and sustainable participation in working life in terms of returning to and staying at work. In 2023, the federal GPI carried out 993,775 medical rehabilitation measures (80% inpatient, 20% outpatient) [3].

During MR, the rehabilitation team determines the individual need for aftercare and recommends specific, suitable measures. According to the Scientific Use File of the GPI (reporting period 2014 to 2021), 31.4% of patients received recommendations for follow-up measures such as rehabilitation sports or functional training [4]. The GPI itself offers several structured aftercare programs for MR patients such as IRENA (Intensivierte Reha-Nachsorge, Intensified Rehabilitation Aftercare) [5–8], which are also available as digital programs [9, 10]. In 2023, about 29% of patients who underwent inpatient MR made use of these programs [3]. Patients can also independently search for aftercare services such as physical therapy practices or outpatient rehabilitation centers offered by GPI-certified or approved facilities [11].

General practitioners (GPs) play an important role in the care and reintegration process following rehabilitation [12]. This includes medical follow-up treatment, but also the coordination of rehabilitation aftercare services as well as motivating patients to use them or to implement the recommendations of the rehabilitation facility. The GP setting allows for the use of communicative, motivational and behavior change techniques, such as goal setting [13], motivational interviewing [14, 15], shared decision-making [16, 17] or other short interventions [18, 19]. These approaches can promote the initiation or continuation of aftercare.

In the German healthcare system, GPs receive a detailed discharge report from the MR facility, which may also contain aftercare recommendations and can serve as a starting point in consultations with the patient. Some German studies have shown that patients see GPs as an important point of contact for MR [20] and want GP support in follow-up care [21]. GPs are generally willing to participate in this process [22].

To date, there have been few studies on how GPs in Germany can be actively involved in improving rehabilitation aftercare in routine care. A pilot study found that when GPs discussed exercise diaries with their patients, the patients were able to increase their physical activity. However, aftercare did not improve outcomes such as functional capacity and participation beyond the effects of rehabilitation [23]. The implementation was rather complex (training of practices, five planned GP contacts and nine telephone calls per patient over 12 months), which raises the question of whether the goal of involving GPs could possibly also be achieved with simpler measures that would be feasible in routine care.

Another pilot study found that the involvement of GPs in the entire rehabilitation process led to an increase in GPs’ awareness of MR and, in particular, to a faster recognition of patients’ rehabilitation needs with an earlier initiation of MR [24]. Since GPs themselves indicate a high need for information on MR and rehabilitation aftercare [25], this implies that greater involvement and participation could encourage GPs to become more active (and better informed) in this area in the long term.

A recent study evaluating a GP-based health check for employees aged 45 and over found an increase in needs-based identification and applications for rehabilitation and preventive services, underlining the important role that GPs can play in this context [26].

## Methods

### Aims

Against this background, we initiated a study to investigate how GPs can be more sustainably involved in aftercare following medical rehabilitation in a structured and low-threshold manner. We will investigate whether a singular, structured follow-up consultation by GPs four to eight weeks after MR contributes to a higher utilization of rehabilitation aftercare by patients who have completed an inpatient MR program. The follow-up consultation will focus on supporting the use of aftercare services based on the rehabilitation facility’s aftercare recommendations listed in the rehabilitation discharge report, discussing obstacles to implementing these recommendations, and identifying patients’ unmet aftercare needs.

The recommendations aim to support GPs in structuring subsequent treatment and thus promote the long-term effectiveness of MR. To support GPs, we will develop a written consultation guide for follow-up consultations based on the information provided in the MR discharge report. In addition, we will develop a corresponding communication aid for patients to help them address questions about aftercare and related support needs during the follow-up consultation, thereby promoting their activation and involvement.

We will also investigate whether the GP follow-up examination is associated with better self-reported work ability. This can be seen as an indicator of effective rehabilitation, as the aim of the GPI’s medical rehabilitation is to secure or restore the ability to work [27]. It has been used as an outcome parameter in various rehabilitation studies [28–34].

Furthermore, we will explore to what extent the intervention is feasible in routine care from the perspective of GPs and how they evaluate it. Finally, we will examine whether the intervention can encourage GPs to engage more intensively with MR in order to better understand its potentials and support patients (e.g., by providing information about MR or assisting in the application process).

We will address the following research questions in a cluster randomized crossover trial:

#### Primary research question

1. Does participation in a structured follow-up consultation with the general practitioner lead to increased utilization (initiation, continuation) of rehabilitation aftercare measures or programs by patients four months after completion of MR?

#### Secondary research questions

2. Is the follow-up consultation associated with a better self-reported work ability four months after completion of MR?
3. Is a structured follow-up consultation feasible as a low-threshold intervention in the GP practice?
4. Can a structured follow-up consultation help to improve the level of information available to GPs on the subject of medical rehabilitation and rehabilitation aftercare?

The study will be conducted as a pragmatic cluster randomized crossover trial using a mixed-methods approach. Study participants will include medical rehabilitation patients and general practitioners. The study is conceived as a superiority trial where the intervention is tested against care-as-usual.

The study protocol was written in accordance with the Standard Protocol Items: Recommendations for Interventional Trials (SPIRIT) statement [35]; additional files 1 and 2.

### Intervention

The intervention addresses MR patients and comprises a one-off follow-up consultation by GPs for medical rehabilitation aftercare four to eight weeks after completion of MR. The consultation lasts (at least) 20 min and is remunerated as part of the project in accordance with No. 34 of the German Medical Fee Index (approx. €40). There are no mandatory appointments for patients with their GP to discuss the discharge report or aftercare recommendations. As such, it is not a “regular” appointment immediately after completion of MR, which patients use, for example, to clarify formal issues (e.g. sick note).

During the consultation, in addition to clarifying the patient’s current health status, the GP will discuss with the patient which of the aftercare measures recommended during MR have already been initiated (e.g., to what extent an IRENA program or a rehabilitation sports program has been started). Regarding possible obstacles to accessing or continuing aftercare, the GP can provide targeted motivation and support, e.g. by identifying or arranging services close to the patient’s home.

The consultation is semi-structured and based on a written consultation guide for GPs, which will be developed in the first study phase. The consultation guide will refer to the rehabilitation discharge report and cover the following topics: patients’ rehabilitation goals; difficulties or obstacles in achieving these goals; aftercare recommendations given during MR; implementation of aftercare recommendations by the patient; experiences including possible obstacles; self-efficacy in implementing/continuing aftercare; desired support/supervision from the GP during aftercare. In developing the consultation guide, we draw on the 5A’s model [36], motivational interviewing, and the taxonomy of behavior change techniques [37, 38] to select practical and evidence-based elements.

A written patient communication aid will be developed to support patients in the follow-up consultation. Where appropriate and useful, it will reflect the content and structure of the GP consultation guide. The communication aid is intended to help patients reflect on the following topics: current state of health; perceived changes and improvements as a result of MR; achievement of rehabilitation goals; implementation of aftercare recommendations. It is designed to encourage patients to ask questions and express their preferences during the follow-up consultation based on the modified PACE framework [39, 40], and to clarify their health-related values [41, 42].

Intervention group participants will receive a written invitation to the follow-up consultation. In order to increase the commitment and attractiveness of the follow-up consultation, the invitation will be communicated as a ”Let’s talk about your health” voucher (including the patient communication aid). The specific distribution of the invitation and accompanying materials depends on when and by whom the follow-up recommendations are provided in the respective rehabilitation facilities, for example, during the final medical consultation. This will be clarified in the first study phase.

### Eligibility criteria

#### Rehabilitation patients

Adult patients aged 18 to 60 years insured with the German Pension Insurance North Bavaria who undergo a medical rehabilitation program in one of six cooperating inpatient rehabilitation centers^1^ during the recruitment period are eligible to participate in the study. All medical indications covered by these facilities are included, with the exception of oncology, psychosomatics, addictive disorders, and rehabilitation measures for children and adolescents. Other exclusion criteria are age under 18 or over 60 years, AHB procedure (“Anschlussrehabilitation”; medical rehabilitation immediately after hospitalization, e.g., following myocardial infarction or surgery), severe cognitive impairment, lack of understanding of the German language.

The same inclusion and exclusion criteria for participant recruitment apply to the cognitive interviews with rehabilitation patients to evaluate the patient communication aid.

#### General practitioners

Eligible for participation in the intervention (follow-up consultation) are GPs (including family doctors specializing in internal medicine) in the northern part of the federal state of Bavaria (administrative districts: Lower Franconia, Middle Franconia, and Upper Franconia) whose patients are undergoing medical rehabilitation at one of the cooperating rehabilitation centers and have agreed to participate in the study.

Eligible participants for the evaluation of the intervention are GPs (incl. GPs in internal medicine) in the Northern part of the federal state of Bavaria (administrative districts: Lower Franconia, Middle Franconia, Upper Franconia) who have participated in the intervention (collection of quantitative evaluation data via online questionnaires).

During the development of the consultation guide for GPs (collection of qualitative data in interviews), GPs (incl. GPs in internal medicine) in the city and surrounding district of Würzburg will be approached.

### Study design and data collection

#### Rehabilitation patients

In the first phase of the study, we will collect qualitative data in interviews with MR patients to evaluate comprehensibility and user-friendliness of the patient communication aid, which is intended to support study participants during the follow-up consultation with their GP. Participants will be presented with the draft version of the communication aid. In all sections, respondents will be asked to explain to what extent the individual items are comprehensible and whether there are any suggestions for rewording or other modifications. The procedure is based on the cognitive interview methodology and the “think aloud” method [43, 44].

In the second study phase, we will collect quantitative data (written questionnaires) in six inpatient rehabilitation centers in Northern Bavaria and neighboring regions. Questionnaire data will be collected longitudinally at three measurement points (T1: end of MR; T2: after follow-up consultation with GP (intervention group only; four to eight weeks after completion of MR); T3: four months after completion of MR).

The primary outcome is the self-reported utilization (initiation/continuation) of rehabilitation aftercare at T3 (four months post-rehabilitation). Self-reported work ability at T3 is assessed as a secondary outcome. We will collect additional variables to describe the sample and/or include them in moderator or predictive analyses. Table 1 summarizes the instruments used at each measurement point.

**Table 1:** Instruments used in the study

Variable	Measure	T1 Completion of MR		T2 After follow-up consultation (IG only)		T3 4 months after completion of MR	
		IG	CG	IG	CG	IG	CG
Rehabilitation aftercare motivation, expectations	single items developed for this study	**X**	**X**				
Rehabilitation aftercare self-efficacy	single items developed for this study	**X**	**X**				
Rehabilitation aftercare recommendations by MR facility	single items developed for this study	**X**	**X**				
Evaluation of medical rehabilitation outcomes (subjective success of rehabilitation)	single items developed for this study	**X**	**X**				
Self-reported work ability	WAS [45, 46]	**X**	**X**				
Health-related quality of life	EQ-5D 5 L [47]	**X**	**X**				
Health literacy	HLS19-Q12-DE [48]	**X**	**X**				
Perceived communicative self-efficacy in patient-physician interactions	Single item developed for this study, based on Maly et al. [49]	**X**	**X**				
Sociodemographic, socio-medical data	single items based on recommended sociodemographic indicators in rehabilitation research [50, 51]	**X**	**X**				
Rehabilitation aftercare motivation	single items developed for this study			**X**	**---**		
Rehabilitation aftercare self-efficacy	single items developed for this study			**X**	**---**		
Use of/participation in follow-up consultation	single items developed for this study			**X**	**---**		
Evaluation of follow-up consultation	single items developed for this study			**X**	**---**		
Implementation of aftercare recommendations by MR facility	single items developed for this study			**X**	**---**		
Evaluation of medical rehabilitation outcomes (subjective success of rehabilitation)	single items developed for this study			**X**	**---**		
Self-reported work ability	WAS [45, 46]			**X**	**---**		
Health-related quality of life	EQ-5D 5 L [47]			**X**	**---**		
Health literacy	HLS19-Q12-DE (51)			**X**	**---**		
Socio-medical data (return to work status)	single items based on recommended sociodemographic indicators in rehabilitation research [50, 51]			**X**	**---**		
Rehabilitation aftercare initiation/ continuation (primary outcome); reasons for non-use	single items developed for this study					**X**	**X**
Rehabilitation aftercare motivation	single items developed for this study					**X**	**X**
Rehabilitation aftercare self-efficacy	single items developed for this study					**X**	**X**
Evaluation of medical rehabilitation outcomes (subjective success of rehabilitation)	single items developed for this study					**X**	**X**
Self-reported work ability	WAS [45, 46]					**X**	**X**
Health-related quality of life	EQ-5D 5 L [47]					**X**	**X**
Health literacy	HLS19-Q12-DE [48]					**X**	**X**
Socio-medical data (return to work status)	single items based on recommended sociodemographic indicators in rehabilitation research [50, 51]					**X**	**X**

Note: MR = medical rehabilitation; IG = intervention group; CG = control group

Given the exploratory nature of the study, we will develop several items specifically for this purpose. These relate to self-reported use (initiation/continuation) of aftercare (primary outcome) as well as the following variables: aftercare motivation; aftercare self-efficacy; list of aftercare recommendations provided by the MR facility (and their implementation); utilization and evaluation of follow-up consultation; evaluation of MR outcomes; perceived communicative self-efficacy in patient-physician interactions (the latter based on Maly et al. [49]).

Self-reported work ability will be assessed by the Work Ability Score (WAS), which is the item “current work ability compared with the lifetime best“ from the Work Ability Index (WAI, German version) [45]. Higher values on a scale ranging from 0 to 10 indicate a better subjective ability to work. The WAS has been used in various studies and has shown satisfactory reliability and validity [46, 52].

Health-related quality of life will be assessed using the German version of the EQ-5D 5 L, an established instrument in healthcare and rehabilitation research with good psychometric properties [47, 53]. It consists of five items that measure different dimensions of health and functioning (mobility, self-care, usual activities, pain/discomfort, anxiety/depression) with a five-point response format, as well as a visual analogue scale to rate the current state of health (from 0 = worst imaginable health to 100 = best imaginable health).

Health literacy will be assessed by the HLS_19_-Q12-DE [48], which is based on the HLS-EU conceptual model of health literacy [54]. It consists of 12 items using a four-point response format (1 = very difficult, 2 = difficult, 3 = easy, 4 = very easy). Evidence from multiple countries supports the instrument’s acceptable reliability and validity [48].

Basic sociodemographic and socio-medical data (incl. return to work status) will be assessed based on published recommendations from rehabilitation research [50, 51].

#### General practitioners

To discuss a draft consultation guide for the intervention, we will collect qualitative data in interviews with GPs. The consultation guide serves as the basis for the structure of the interviews. No further structuring is used in order to obtain a broad range of feedback and comments, which will be incorporated into a revision of the guide.

We will collect quantitative data using a short online questionnaire that GPs will complete after each follow-up consultation. The questionnaire will assess the consideration and use of the rehabilitation discharge report for the consultation, the planning of further treatment and communication with the patient in this regard, as well as the evaluation of the aftercare recommendations of the rehabilitation facility.

Additionally, toward the end of the intervention and data collection, GPs will complete a one-time online survey to evaluate the intervention in summary. The online survey will assess the following variables:

- Overall evaluation of the follow-up consultation (feasibility, motivation of patients).
- Use of the consultation guide (to check for treatment integrity), evaluation of the consultation guide.
- Evaluation of rehabilitation outcomes and effectiveness (in terms of “rehabilitation success”).
- Level of information and information needs regarding MR and rehabilitation aftercare.

We will develop items specifically for this purpose.

**Fig. 1:**
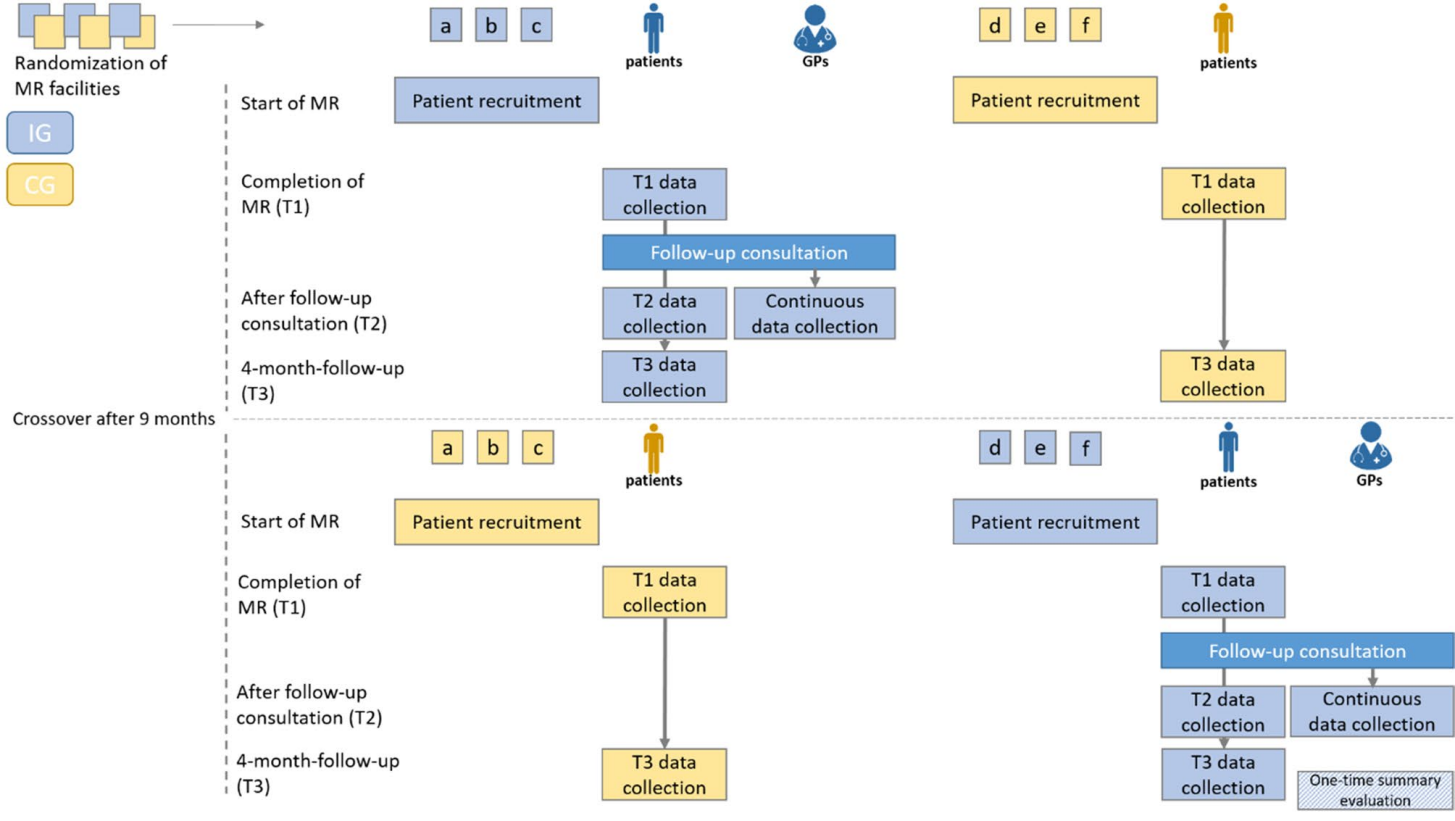
Timeline of data collection. Note: MR = medical rehabilitation; IG = intervention group; CG = control group; GPs = general practitioners. Interviews with patients or GPs (study phase 1) are not shown

Figure 1 shows the data collection timeline.

The SPIRIT flow diagram in additional file 3 summarizes the timeline for enrolment, interventions and assessments.

### Randomization

**Fig. 2:**
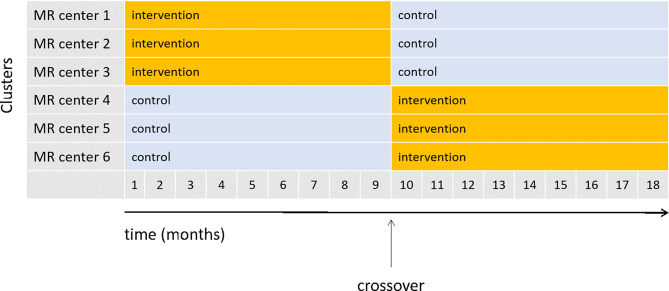
Crossover design. Note: MR = medical rehabilitation

Using a two-period cluster randomized crossover design, study participants (rehabilitation patients) will be randomized at the facility level: during the first nine months of data collection, the intervention will be carried out in half of the cooperating rehabilitation centers (clusters), i.e., all study participants in these facilities are assigned to the intervention condition. In the other half of clusters, the control condition is implemented. After nine months, the clusters cross so that the intervention condition will now be implemented in the previous “control clusters” and vice versa (Fig. 2). Which facilities enter the intervention or control phase first is determined in advance by randomization.

Participants in the intervention group (IG) and the control group (CG) will receive the usual recommendations for rehabilitation aftercare during their rehabilitation stay. Participants in the IG will also receive an invitation for the follow-up consultation with their GP, which is scheduled to take place four to eight weeks after completion of MR. Participants in the control group (CG) do not receive a follow-up consultation with their GP.

Given the small scale and effort of the intervention required on the part of the rehabilitation centers, we do not expect any carry-over effects and see no need to introduce a washout period before the change of conditions in the facilities [55].

The study staff will randomly assign the clusters using an online research tool (Research Randomizer; randomizer.org).

There will be no blinding of healthcare professionals in the rehabilitation centers or GPs, as knowledge of the status of study participants (intervention / control condition) is necessary for the provision of information and materials (rehabilitation centers) or the intervention (GPs). The study team will inform the rehabilitation centers of their respective allocation in written form so that they can organize study materials and procedures accordingly.

On the part of rehabilitation patients, study participants will be blinded with regard to group assignment (intervention/control condition). Due to the nature of the study, we do not expect that emergency unblinding will be necessary.

### Sample size

#### Rehabilitation patients

We expect an effect of the intervention on patients’ self-reported use of rehabilitation aftercare four months after they have completed MR (primary outcome). Specifically, we assume that more patients in the IG will use or continue to use aftercare services than patients in the CG. Since CG patients also receive follow-up recommendations from their MR facility, we expect a small effect in favor of the IG.

Utilization of the GPI aftercare programs (IRENA, other) is around 30% [3, 56], which can be used as a minimum approximation for the control condition. We assume an additional utilization rate of 10% as a result of the intervention, i.e. 40% in total. This estimate is exploratory and, given the lack of data, rather conservative.

To calculate the sample size for the cluster randomized crossover trial, we used the Shiny CRT Calculator program [57, 58]. Our estimate for the intra-cluster correlation (ICC) is based on results from a study using GPI routine data for various medical indications (*n* = 653,081) [59]. In this study, researchers developed a risk adjustment strategy to improve the use of return to work as a quality indicator for medical rehabilitation. The ICC for the rehabilitation department level (i.e. indications) averages 1.95% (0.0195).

For six cooperating facilities (clusters), a sample size of 29 participants per cluster (clinic) and time period (intervention/control phase; see Fig. 1) is calculated with a power of 0.8 to demonstrate a small intervention effect, i.e. 174 persons for both the intervention and control conditions (total *n* = 348).

We calculated an expected dropout of approximately 35% from T1 to T2. Due to a lack of data from comparable studies, we used willingness to participate in various medical rehabilitation or aftercare studies as a proxy for willingness to participate in the follow-up consultation [23, 60–68]. Willingness to participate in these studies ranged between 42% and 85% (mean: 67%). Moreover, presumably not all study participants will take part in the follow-up consultation and its use is also contingent upon the participation of the respective GPs. On this basis, we assume a realistic dropout rate of 35%.

From T2 to T3, a further dropout of approximately 20% can be assumed. Data from various rehabilitation studies with 3- or 6-month follow-ups show dropout rates ranging between 5% and 28%, with an average of 14% [30, 32, 65, 69–76]. A conservative estimate is therefore a dropout of 20%. This increases the required sample size to a total of *N* = 669 individuals (T1) (T2: *n* = 435 due to a dropout rate of 35%).

The aim of the interviews with rehabilitation patients to evaluate the communication aid for patients (Phase 1) is to cover all indications included in the cooperating rehabilitation centers (orthopedics, endocrinology/diabetology, cardiology). We plan to recruit two to three individuals per indication. The objective of the interviews has a defined focus, the sample can be considered specific to the study context, and the interviews are characterized by a high degree of interactivity and dialogue. According to Malterud et al. [77], it can therefore be assumed that the proposed number of participants is sufficient to obtain the desired information for evaluating the communication aid. In addition, this limits the effort required to conduct the interviews (recruiting participants by clinic staff, finding time slots for participants outside their therapy schedules, organizing and securing rooms in the rehabilitation center, travel arrangements for project staff).

#### General practitioners

For the group of general practitioners, the following estimates of the number of participants can be made:

The number of GPs in the study region among whom the potential study participants (patients) are distributed (i.e. how many GP practices would need to be included) can only be roughly estimated. There are 3,050 GPs in Northern Bavaria [78]. There is no care or billing data available on how many of the patients receiving primary/GP care are currently employed or at least 18 years old and thus eligible to participate in the study. Therefore, we cannot rely on specific proportions or frequencies here.

The starting point for estimating the sample size is the following data: The GPI North Bavaria provided a total of 34,513 medical rehabilitation measures in 2024 and 10,096 aftercare services in the form of IRENA, T-RENA (Trainingstherapeutische Rehabilitationsnachsorge; Training/Exercise Therapy Rehabilitation Aftercare), and Psy-RENA (Psychosomatische Rehabilitationsnachsorge; Psychosomatic Rehabilitation Aftercare) [56]. This means that about 30% of patients make use of such aftercare services. Assuming that an additional 10% of patients use aftercare as a result of the intervention (i.e. a total of 40%, corresponding to *n* = 13,805), based on the estimated number of cases for rehabilitation patients (see above, *n* = 348), *n* = 77 GPs in northern Bavaria would be required as study participants to carry out the intervention.

The interviews with GPs during the development of the consultation guide will comprise a sample size of 8 to 10 participants. This should reflect a sufficient spectrum in terms of medical practice size and the number of patients.

The following considerations regarding sample size are used for the online survey to answer the secondary research questions (evaluation of the intervention concept; increase in knowledge of GPs regarding MR and rehabilitation aftercare). An online survey of GPs on medical rehabilitation in the federal state of Schleswig-Holstein had a participation rate of 27.7% (*n* = 194), based on the total number of respondents in the mailing list of the Schleswig-Holstein Association of Statutory Health Insurance Physicians (*n* = 700) [25]. Based on this percentage and the estimates presented above, we assume that 21 GPs will participate in the online survey. It is possible that the willingness to complete the questionnaire is higher due to participation in the study, which would result in a larger proportion of participating physicians.

### Recruitment

In each cooperating rehabilitation center, a responsible person (“study coordinator”) will be appointed, who will be briefed by the research team and be provided with all important information, including inclusion and exclusion criteria, as well as materials for recruitment and data collection (study information, declaration of consent, questionnaires) within the facility. This person will be specifically responsible for contacting eligible patients and asking them to participate in the study, distributing (and collecting) all relevant documents after informed consent to participate in the study has been given, and providing and managing materials related to the intervention (information flyers/vouchers for the follow-up consultation) during the data collection period in which the respective facility is assigned to the intervention condition.

The rehabilitation center will hand out T1 questionnaires to study participants. To ensure anonymity, the respective rehabilitation centers will also mail T2 and T3 questionnaires to study participants (the latter can also be completed online). To simplify their return and to reduce the dropout rate, the envelopes for all questionnaires (T1, T2, T3) are prepaid. The rehabilitation facilities will receive a flat fee of €20 per study participant recruited for their recruitment efforts.

For practical reasons, we will recruit GPs via various channels:

First, the Bavarian Association of General Practitioners will inform GPs about the study (incl. eligibility for reimbursement of follow-up consultations) via the association’s newsletter and other resources, several times if necessary.

In addition to the rehabilitation discharge report, which is sent to the respective GP as standard, the cooperating rehabilitation centers will enclose an information sheet and other written materials that identify the respective patient as a study participant.

The sheet contains information about the study and the follow-up consultation. In particular, it includes a link to the project website, where GPs will complete the evaluation questionnaire after each follow-up consultation.

Study patients will also receive an “information package” containing the relevant study documents as well as information and materials for their GP. When making an appointment for the follow-up consultation, patients will be asked to present the materials to their GP and ask them to participate in the study.

For the interviews with rehabilitation patients in the first study phase, the study coordinators at the cooperating rehabilitation centers will approach potentially eligible patients based on the inclusion criteria (see above) and ask them to participate in the interviews. Once consent to participate has been obtained, an appointment will be scheduled at the rehabilitation center. Whether interview participants will be recruited in all rehabilitation centers or only in some of them will be decided depending on the respective resources and possibilities on site.

For the interviews with GPs in the first study phase, we will contact general practitioners in the Würzburg area whose practices are part of the local network of teaching practices of the Department of General Practice at the University of Würzburg and ask for participation.

### Retention strategies

We consider the following procedures as important in order to achieve a high participation rate among patients: The questionnaires for study participants are pre-franked to facilitate return mailing. The study team will design the questionnaires as short as possible so that the effort for participants to complete them is relatively low. The invitation to participate in the study comes from the rehabilitation facility, which patients are likely to view as a trustworthy institution. Recruitment will focus on the benefits of the intervention in terms of an additional, low-threshold offer for patients provided by their GP.

No concomitant care or interventions are prohibited during the conduct of the study.

To ensure treatment integrity at the facility level, the organizational processes (recruitment, data collection, etc.) will be communicated uniformly and thus standardized.

The following procedures are intended to encourage GPs to participate in the study: The intervention will be reimbursed as a medical service. The intervention and study participation are supported and endorsed by the Bavarian Association of General Practitioners and the Bavarian Association of Statutory Health Insurance Physicians. GPs will be provided with tools to help them implement the intervention (consultation guide, easily accessible billing mode).

### Statistical methods, data analysis

To investigate the effect of the intervention on self-reported use of rehabilitation aftercare at T3 (primary outcome), analysis of variance and related methods (analysis of covariance, regression analysis) will be used. We will conduct moderator analyses to examine the potential moderating role of variables (e.g., age, sex, health literacy level) and to analyze potential subgroups.

Descriptive analyses (means, standard deviations, correlation analyses) and qualitative analyses will be used to answer the secondary research questions.

IBM SPSS version 29 will be used for statistical analyses.

To assess potential dropout bias, we will perform subgroup analyses comparing complete datasets with those containing missing data. If the missing data shows to be Missing At Random (MAR), we plan to apply multiple imputation techniques to increase the statistical power and robustness of our analyses.

### Data management

All measures for data collection, processing, protection, and IT security have been recorded in detail in a written data protection concept, which has been submitted to the Data Protection Officer of the German Pension Insurance North Bavaria (Deutsche Rentenversicherung Nordbayern) for review and approval.

Collected paper questionnaires from patients will be scanned, the digitized data from questionnaires will be stored in encrypted form on servers of the University Hospital Würzburg.

The collection and encrypted storage of online questionnaire data from GPs is carried out on servers of the University Hospital Würzburg. A possible alternative is the “SoSciSurvey” service, whose servers are operated in Germany and which enables GDPR-compliant data collection.

Paper questionnaires and online questionnaires are pseudonymized using an alphanumeric ID number.

Qualitative data collected in interviews (audio files) will be transcribed, the transcripts will then be stored on University Hospital servers. The interview transcripts are each assigned an alphanumeric code (pseudonymization) and any personal data is removed.

All data is stored in password-protected directories that can only be accessed by the study team.

Trained student research assistants and/or researchers from the study team will be responsible for data entry and verification.

The original data will be stored for a period of 10 years after the end of the study.

No interim analyses are planned.

We do not expect harmful or adverse events due to the nature of the study. Accordingly, we did not specify criteria for discontinuing or modifying the intervention for specific study participants.

### Data monitoring

A data monitoring committee is not required for the study, as is it not a clinical trial or testing of a therapeutic intervention, medical procedure or drug. Neither is an endpoint adjudication committee needed, as this study does not investigate any pharmacological or other clinical/therapeutic intervention.

No auditing for trial conduct is planned in this study.

Due to the scope and nature of the study, no separate steering committee is established.

The research, study coordination, and data management team within the Rehabilitation Sciences Section at the University Hospital Würzburg consists of the authors of the protocol. The study team has the statistical and methodological expertise to conduct the study. Trained student assistants will provide support with data entry and verification.

### Dissemination

Study results will be published in peer-reviewed journals and presented at scientific conferences.

If the study confirms the hypothesized effectiveness of the intervention, we will make the procedures and materials (e.g., consultation guide, patient communication aid) available to healthcare professionals. A dissemination could be carried out via associations of general practitioners/family doctors, health organizations or other relevant stakeholders. All members of the study team will jointly prepare publications, including the final study report.

## Discussion

The aim of the study is to evaluate an intervention in which GPs support patients after medical rehabilitation in continuing health-promoting behaviors, skills, and treatment learned during rehabilitation.

Given the high workload of GPs, the intervention is designed to be low-threshold to ensure its feasibility. It is limited in scope (follow-up consultation) and accompanied by supporting tools to help GPs implement the intervention (consultation guide, easy to access billing mode). Patient-centered materials will also be developed to support patients in utilizing the intervention delivered by their GP (patient communication aid), as patients consider GPs to be a trusted source of information regarding medical rehabilitation [20, 25].

The intervention can help raise awareness among GPs of the potentials of medical rehabilitation. This is important given the gaps in knowledge and information needs that GPs in Germany themselves report with respect to medical rehabilitation [25, 79]. A better understanding of rehabilitation by GPs may contribute to the sustainability of rehabilitation by better aligning further treatment with the recommendations from rehabilitation or by supporting their patients in integrating the results of rehabilitation (including disease management/self-management skills and lifestyle changes) into their own everyday lives. GPs would also be better able to inform other patients about rehabilitation and to support them, for example, in applying for rehabilitation.

By supporting transition in care or bridging sectoral interfaces, GPs can contribute to the coordination and continuity of healthcare, which is a conducive prerequisite for patient-centered care [80]. Compared to countries where rehabilitation is mainly provided on an outpatient basis, the transfer of rehabilitation outcomes into everyday life may be more challenging in Germany due to the transition from inpatient treatment to the use of aftercare services at home as an interface that needs to be managed. GPs play an important role in this regard. On the other hand, given the specific characteristics of the German (or any other) national healthcare system, the intervention is context-specific, and its adaptation to other contexts or care pathways would require modifications.

The motivation of participating GPs could be a possible confounder, as GPs who consider medical rehabilitation and follow-up care to be effective and relevant are likely to conduct their consultations differently to those who do not. This issue will be addressed by randomizing patients at the cluster level (i.e., rehabilitation clinics) and by providing a structured consultation guide. In addition, the online survey of GPs will also contain questions about their level of information and attitudes towards medical rehabilitation and aftercare.

The degree to which participating GPs implement the intervention (using the consultation guide) will be assessed using relevant items in the online survey and is also indirectly documented via the patient survey. As mentioned above, the organizational procedures (recruitment, data collection, etc.) are communicated to the participating rehabilitation centers in a standardized way to ensure treatment integrity at the facility level.

## Electronic Supplementary Material

Below is the link to the electronic supplementary material.

Additional file 1: SPIRIT_checklist

Additional file 2: WHO Trial Registration Data Set

Additional file 3: SPIRIT flow diagram

## Data Availability

Data are not available as the participants of this study do not give written consent for their data to be shared publicly. Summarized (aggregated) results will be presented at research conferences and in peer-reviewed articles.

## Footnotes

1 A list of the study sites can be obtained from the authors upon request.

## Abbreviations

CG: Control group
DRKS: Deutsches Register Klinischer Studien (German Clinical Trials Register)
DRV: Deutsche Rentenversicherung (German Pension Insurance)
ICC: Intra-cluster correlation
IG: Intervention group
IRENA: Intensivierte Reha-Nachsorge (Intensified Rehabilitation Aftercare)
MR: Medical rehabilitation
Psy-RENA: Psychosomatische Rehabilitationsnachsorge (Psychosomatic Rehabilitation Aftercare)
SPIRIT: Standard Protocol Items: Recommendations for Interventional Trials
T-RENA: Trainingstherapeutische Rehabilitationsnachsorge (Training/Exercise Therapy Rehabilitation Aftercare)

## References

[1] World Health Organization (WHO). The International Classification of Functioning, Disability and Health. 2001. http://www.who.int/classifications/icf/en/. Accessed 5 November, 2025.

[2] Mittag O, Welti F. Medizinische Rehabilitation im europäischen Vergleich und Auswirkungen des europäischen Rechts auf die deutsche Rehabilitation [Comparison of medical rehabilitation in various European countries and the impact of European law on rehabilitation practice in Germany]. Bundesgesundheitsblatt Gesundheitsforschung Gesundheitsschutz. 2017;60(4):378–85. 10.1007/s00103-017-2516-y. 28224186 10.1007/s00103-017-2516-y

[3] Deutsche Rentenversicherung Bund (DRV Bund). Reha-Bericht 2024 [Rehabilitation Report 2024]. 2024. https://www.deutsche-rentenversicherung.de/SharedDocs/Downloads/DE/Statistiken-und-Berichte/Berichte/rehabericht_2024.html. Accessed 5 November, 2025.

[4] Forschungsdatenzentrum der Rentenversicherung (FDZ-RV). Scientific Use File - Abgeschlossene Rehabilitation im Versicherungsverlauf 2014–2021 [Scientific Use File - Completed rehabilitation measures in the insurance history 2014–2021]. Berlin. 10.57701/SUF.RSDV.2021.1-0.

[5] Lamprecht J, Behrens J, Mau W, Schubert M. Das Intensivierte Rehabilitationsnachsorgeprogramm (IRENA) der Deutschen Rentenversicherung Bund - Berufsbegleitende Inanspruchnahme und Veränderungen berufsbezogener Parameter [Intensified rehabilitation aftercare (IRENA): utilization alongside work and changes in work-related parameters]. Rehabilitation. 2011;50(3):186–94. 10.1055/s-0031-1275688. 21626466 10.1055/s-0031-1275688

[6] Lamprecht J, Behrens J, Mau W, Schubert M. Das Intensivierte Rehabilitationsnachsorgeprogramm (IRENA) der Deutschen Rentenversicherung Bund: Therapiegeschehen und Ein-Jahres-Verlauf gesundheitsbezogener Parameter bei Rehabilitanden mit muskuloskelettalen Erkrankungen [Intensified Rehabilitation Aftercare (IRENA) of the German Pension Insurance Fund – therapy and the one-year-course of health related parameters in orthopedic patients]. Phys Rehab Kur Med. 2012;22(05):253–7. 10.1055/s-0032-1314860.

[7] Schilling G. Was kommt nach der Rehabilitation? Schnittstelle Reha – Nachsorgeprogramme [What comes after rehabilitation? Interface between rehabilitation and aftercare programs]. Rehabilitation. 2023;62(04):195–6. 10.1055/a-2118-9936.

[8] Deutsche Rentenversicherung Bund (DRV Bund). Rahmenkonzept zur Reha-Nachsorge der Deutschen Rentenversicherung [Framework concept for medical rehabilitation aftercare of the statutory pension insurance]. 2019. https://www.deutsche-rentenversicherung.de/SharedDocs/Downloads/DE/Experten/infos_reha_einrichtungen/konzepte_systemfragen/konzepte/rahmenkonzept_reha_nachsorge.html. Accessed 5 November, 2025.

[9] Jekauc D, Rayling S, Klopp S, Schmidt D, Rittmann L-M, Fritsch J. Effects of a web-based rehabilitation aftercare on subjective health, work ability and motivation: a partially randomized controlled trial. BMC Musculoskelet Disord. 2021;22(1):366. 10.1186/s12891-021-04239-z. 33874917 10.1186/s12891-021-04239-z PMC8054846

[10] Schmidt D, Hedin J, Pelegrina A, Weyland S, Rittmann L-M, Jekauc D. Comparing the effectiveness of digital and conventional rehabilitation aftercare on work ability in orthopedic patients: A longitudinal study in Germany. J Occup Rehabil. 2025. 10.1007/s10926-025-10284-5. 40146406 10.1007/s10926-025-10284-5 PMC13099794

[11] Parzanka S, Himstedt C, Deck R. Entwicklung eines konsentierten Kriterien-Sets zur Bewertung von Reha-Nachsorgeangeboten [Development of a consented set of criteria to evaluate post-rehabilitation support services]. Z Evid Fortbild Qual Gesundhwes. 2015;109(8):578–84. 10.1016/j.zefq.2015.09.028. 26704819 10.1016/j.zefq.2015.09.028

[12] European Agency for Safety and Health at Work (EU-OSHA). Rehabilitation and return to work: Analysis report on EU and Member States policies, strategies and programmes. 2016. https://osha.europa.eu/sites/default/files/rehabilitation-and-return-to-work-analysis-report-on-eu-and-member-states-policies-strategies-and-programmes.pdf. Accessed 5 November, 2025.

[13] Boeykens D, Boeckxstaens P, an de Sutter, Lahousse L, Pype P, de Vriendt P, et al. Goal-oriented care for patients with chronic conditions or multimorbidity in primary care: A scoping review and concept analysis. PLoS ONE. 2022;17(2):e0262843. 10.1371/journal.pone.0262843. 35120137 10.1371/journal.pone.0262843 PMC8815876

[14] Morton K, Beauchamp M, Prothero A, Joyce L, Saunders L, Spencer-Bowdage S, et al. The effectiveness of motivational interviewing for health behaviour change in primary care settings: a systematic review. Health Psychol Rev. 2015;9(2):205–23. 10.1080/17437199.2014.882006. 26209209 10.1080/17437199.2014.882006

[15] VanBuskirk KA, Wetherell JL. Motivational interviewing with primary care populations: a systematic review and meta-analysis. J Behav Med. 2014;37(4):768–80. 10.1007/s10865-013-9527-4. 23934180 10.1007/s10865-013-9527-4 PMC4118674

[16] Coronado-Vázquez V, Canet-Fajas C, Delgado-Marroquín MT, Magallón-Botaya R, Romero-Martín M, Gómez-Salgado J. Interventions to facilitate shared decision-making using decision aids with patients in primary health care: A systematic review. Medicine. 2020;99(32):e21389. 10.1097/MD.0000000000021389. 32769870 10.1097/MD.0000000000021389 PMC7593011

[17] Heron N, O’Connor SR, Kee F, Thompson DR, Cupples M, Donnelly M. Refining a primary care shared decision-making aid for lifestyle change: a mixed-methods study. BJGP Open. 2022;6(1). 10.3399/BJGPO.2021.0100. 10.3399/BJGPO.2021.0100 PMC8958746 34853008

[18] Rosembaun A, Rojas P, Rodriguez MV, Barticevic N, Rivera Mercado S. Brief interventions to promote behavioral change in primary care settings, a review of their effectiveness for smoking, alcohol and physical inactivity. Medwave. 2018;18(1):e7148. 10.5867/medwave.2018.01.7148. 29385118 10.5867/medwave.2018.01.7148

[19] Searight HR. Counseling patients in primary care: Evidence-based strategies. Am Fam Physician. 2018;98(12):719–28. 30525356

[20] Golla A, Saal S, Meyer G, Frese T, Mikolajczyk R, Richter M, et al. Verständnis und Bedürfnis medizinischer Rehabilitation in der Bevölkerung – Ergebnisse einer Online-Befragung [Comprehension and perspectives of the need for medical rehabilitation - results of a German online survey]. Rehabilitation. 2023;62(4):197–206. 10.1055/a-1998-6673. 36806190 10.1055/a-1998-6673

[21] Korsch S, Herbold D, Wiezoreck M, Geigner B, Beddies A, Worringen U, et al. Förderfaktoren, Barrieren und Barrierenmanagement zur Umsetzung gesundheitsförderlicher Verhaltensweisen von Rehabilitanden mit chronischem Rückenschmerz - Eine qualitative Analyse [Promoting factors, barriers and barrier management to the implementation of health-promoting behavior among rehabilitative patients with chronic low back pain - a qualitative analysis]. Rehabilitation. 2016;55(4):210–6. 10.1055/s-0042-106844. 27529297 10.1055/s-0042-106844

[22] Träder J-M. Reha-Nachsorge in Hausarztpraxen –quälende Pflicht oder verlockende Chance? [Rehabilitation follow-up care in general practice – a torturous obligation or a tempting opportunity?]. In: Deck R, Glaser-Möller N, editors. Reha-Nachsorge – aktuelle Entwicklungen [Rehabilitation aftercare - current developments]. Lage: Jacobs; 2014. pp. 179–86.

[23] Weier L, Steinhäuser J, Träder J-M, Deck R. Hausarztzentrierte Rehabilitationsnachsorge bei chronischen Rückenschmerzen [General practitioner centered rehabilitation aftercare for chronic back pain]. Rehabilitation. 2021;60(3):195–203. 10.1055/a-1286-2595. 33477195 10.1055/a-1286-2595

[24] Jankowiak S, Kaluscha R, Krischak G. Einbindung des Hausarztes in die Rehabilitationsnachsorge – Effekte auf das subjektive und objektive Behandlungsergebnis [Involvement of the general practitioner in rehabilitation aftercare – effects on subjective and objective treatment outcomes]. DRV-Schriften. 111:207–9.

[25] Walther AL, Pohontsch NJ, Deck R. Informationsbedarf zur medizinischen Rehabilitation der Deutschen Rentenversicherung—Ergebnisse eines Online-Surveys mit niedergelassenen Ärzten [Need for information concerning medical rehabilitation of the federal German pension fund–findings of an online survey of general practitioners]. Gesundheitswesen. 2015;77(5):362–7. . 10.1055/s-0034-1377034 25025292 10.1055/s-0034-1377034

[26] Burchardi JM, Gellert P, Brünger M. A work-related health check to identify the need for rehabilitation and preventive care (Check-Up 45+): A multicenter randomized controlled trial in general practice (PReHa45). Dtsch Arztebl Int. 2025;122:315–20. 10.3238/arztebl.m2025.0055. 40293186 10.3238/arztebl.m2025.0055 PMC12550758

[27] Deutsche Rentenversicherung Bund (DRV Bund). Rahmenkonzept zur medizinischen Rehabilitation in der gesetzlichen Rentenversicherung [Framework concept for medical rehabilitation of the statutory pension insurance]. 2009. https://www.deutsche-rentenversicherung.de/SharedDocs/Downloads/DE/Experten/infos_reha_einrichtungen/konzepte_systemfragen/konzepte/rahmenkonzept_medizinische_reha.html. Accessed 5 November, 2025.

[28] Bethge M, Markus M, Streibelt M, Gerlich C, Schuler M. Effects of nationwide implementation of work-related medical rehabilitation in Germany: propensity score matched analysis. Occup Environ Med. 2019;76(12):913–9. 10.1136/oemed-2019-106065. 31594839 10.1136/oemed-2019-106065

[29] Briest J, Bethge M. Intensivierte medizinisch-beruflich orientierte Rehabilitationsnachsorge: Langfristige Ergebnisse der randomisiert-kontrollierten Multicenter-Studie [Intensified work-related rehabilitation aftercare: Long-term results of a randomized controlled multicenter trial]. Rehabilitation. 2016;55(2):108–14. 10.1055/s-0042-102998. 27070985 10.1055/s-0042-102998

[30] Fauser D, Wienert J, Beinert T, Schmielau J, Biester I, Krüger H-U, et al. Work-related medical rehabilitation in patients with cancer - postrehabilitation results from a cluster-randomized multicenter trial. Cancer. 2019;125(15):2666–74. 10.1002/cncr.32131. 30985930 10.1002/cncr.32131

[31] Markus M, Euhus A, Bethge M. Effectiveness of behavioural medical rehabilitation under real-life conditions in Germany: A propensity-score matched analysis. J Rehabil Med. 2022;54:jrm00248. 10.2340/jrm.v53.469.34672356 10.2340/jrm.v53.469

[32] Rutsch M, Deck R. Berufliche Belastungen von Long-Covid-Rehabilitand*innen und Rückkehr zur Arbeit nach einer pneumologischen Rehabilitation [Occupational stress of Long Covid rehabilitants and return to work after pneumological rehabilitation]. Rehabilitation. 2023;62(6):369–78. 10.1055/a-2105-5810. 37595619 10.1055/a-2105-5810

[33] Waranski M, Garbsch R, Kotewitsch M, Teschler M, Schmitz B, Mooren FC. A Behavioral change-based mobile intervention for promoting regular physical activity in medical rehabilitation maintenance of patients with coronary artery disease: Controlled trial. J Med Internet Res. 2024;26:e56480. 10.2196/56480. 39378432 10.2196/56480 PMC11496926

[34] Wienert J, Bethge M. Medizinisch-beruflich orientierte Rehabilitation für onkologische Rehabilitanden – kurzfristige Ergebnisse einer clusterrandomisierten Multicenterstudie [Work-related medical rehabilitation in cancer rehabilitation - short-term results from a cluster-randomized multicenter trial]. Rehabilitation. 2019;58(3):181–90. 10.1055/a-0604-0157. 29801187 10.1055/a-0604-0157

[35] Chan A-W, Tetzlaff JM, Gøtzsche PC, Altman DG, Mann H, Berlin JA, et al. SPIRIT 2013 explanation and elaboration: guidance for protocols of clinical trials. BMJ. 2013;346:e7586. 10.1136/bmj.e7586. 23303884 10.1136/bmj.e7586 PMC3541470

[36] World Health Organization (WHO). Toolkit for delivering the 5A’s and 5R’s brief tobacco interventions in primary care. 2014. https://iris.who.int/bitstream/handle/10665/112835/9789241506953_eng.pdf. Accessed 5 November, 2025.

[37] Michie S, Richardson M, Johnston M, Abraham C, Francis J, Hardeman W, et al. The behavior change technique taxonomy (v1) of 93 hierarchically clustered techniques: building an international consensus for the reporting of behavior change interventions. Ann Behav Med. 2013;46(1):81–95. 10.1007/s12160-013-9486-6. 23512568 10.1007/s12160-013-9486-6

[38] Carey RN, Connell LE, Johnston M, Rothman AJ, de Bruin M, Kelly MP, et al. Behavior change techniques and their mechanisms of action: A synthesis of links described in published intervention literature. Ann Behav Med. 2019;53(8):693–707. 10.1093/abm/kay078. 30304386 10.1093/abm/kay078 PMC6636886

[39] Cegala DJ, McClure L, Marinelli TM, Post DM. The effects of communication skills training on patients’ participation during medical interviews. Patient Educ Couns. 2000;41(2):209–22. 10.1016/s0738-3991(00)00093-8. 12024545 10.1016/s0738-3991(00)00093-8

[40] D’Agostino TA, Atkinson TM, Latella LE, Rogers M, Morrissey D, DeRosa AP, et al. Promoting patient participation in healthcare interactions through communication skills training: A systematic review. Patient Educ Couns. 2017;100(7):1247–57. 10.1016/j.pec.2017.02.016. 28238421 10.1016/j.pec.2017.02.016 PMC5466484

[41] Armstrong MJ, Mullins CD. Value assessment at the point of care: Incorporating patient values throughout care delivery and a draft taxonomy of patient values. Value Health. 2017;20(2):292–5. 10.1016/j.jval.2016.11.008. 28237212 10.1016/j.jval.2016.11.008 PMC5417067

[42] Cornelissen LE, van der Mark EJ, Pennings P, Maat B, Foekens T, Willemsen-de Mey G, et al. What matters to patients with rheumatoid arthritis when facing medical or non-medical treatment decisions? Patient Prefer Adherence. 2021;15:1827–41. 10.2147/PPA.S322257. 34465982 10.2147/PPA.S322257 PMC8403072

[43] Boren MT, Ramey J. Thinking aloud: reconciling theory and practice. IEEE Trans Prof Commun. 2000;43:261–78. 10.1109/47.867942.

[44] Prüfer P, Rexroth M. Kognitive Interviews [GESIS-How‐to 15] [Cognitive interviews]. 2005. https://nbn-resolving.org/urn:nbn:de:0168-ssoar-201470. Accessed 5 November, 2025.

[45] Hasselhorn HM, Freude G. Der Work Ability Index. Ein Leitfaden [The Work Ability Index - a guide]. Bremen: Wirtschaftsverlag NW Verlag für Neue Wissenschaft; 2007.

[46] Ebener M, Hasselhorn HM. Validation of short measures of work ability for research and employee surveys. Int J Environ Res Public Health. 2019;16(18). 10.3390/ijerph16183386. 10.3390/ijerph16183386 PMC6765804 31547466

[47] Buchholz I, Janssen MF, Kohlmann T, Feng Y-S. A systematic review of studies comparing the measurement properties of the three-level and five-level versions of the EQ-5D. PharmacoEconomics. 2018;36(6):645–61. 10.1007/s40273-018-0642-5. 29572719 10.1007/s40273-018-0642-5 PMC5954044

[48] Pelikan JM, Link T, Straßmayr C, Waldherr K, Alfers T, Bøggild H, et al. Measuring comprehensive, general health literacy in the general adult population: The development and validation of the HLS19-Q12 instrument in seventeen countries. Int J Environ Res Public Health. 2022;19(21). 10.3390/ijerph192114129. 10.3390/ijerph192114129 PMC9659295 36361025

[49] Maly RC, Frank JC, Marshall GN, DiMatteo MR, Reuben DB. Perceived efficacy in patient-physician interactions (PEPPI): validation of an instrument in older persons. J Am Geriatr Soc. 1998;46(7):889–94. 10.1111/j.1532-5415.1998.tb02725.x. 9670878 10.1111/j.1532-5415.1998.tb02725.x

[50] Deck R, Röckelein E. Zur Erhebung soziodemographischer und sozialmedizinischer Indikatoren in den rehabilitationswissenschaftlichen Forschungsverbünden [On the collection of sociodemographic and socio-medical indicators in rehabilitation research networks]. Verband Deutscher Rentenversicherungsträger VDR, editor. Förderschwerpunkt „Rehabilitationswissenschaften - Empfehlungen der Arbeitsgruppen „Generische Methoden, „Routinedaten und „Reha-Ökonomie. DRV-Schriften. 1999;(16):84–98.

[51] Nübling R, Kaluscha R, Krischak G, Kriz D, Martin H, Müller G, et al. Return to Work nach stationärer Rehabilitation – Varianten der Berechnung auf der Basis von Patientenangaben und Validierung durch Sozialversicherungs-Beitragszahlungen [Return to work after inpatient rehabilitation – alternative calculations on the basis of patient data and validation of social security contributions]. Phys Rehab Kur Med. 2016;26(06):293–302. 10.1055/s-0042-117282.

[52] Gragnano A, Villotti P, Larivière C, Negrini A, Corbière M. A Systematic search and review of questionnaires measuring individual psychosocial factors predicting return to work after musculoskeletal and common mental disorders. J Occup Rehabil. 2021;31(3):491–511. 10.1007/s10926-020-09935-6. 33355911 10.1007/s10926-020-09935-6 PMC8298352

[53] Feng Y-S, Kohlmann T, Janssen MF, Buchholz I. Psychometric properties of the EQ-5D-5L: a systematic review of the literature. Qual Life Res. 2021;30(3):647–73. 10.1007/s11136-020-02688-y. 33284428 10.1007/s11136-020-02688-y PMC7952346

[54] Sørensen K, van den Broucke S, Fullam J, Doyle G, Pelikan J, Slonska Z, et al. Health literacy and public health: a systematic review and integration of definitions and models. BMC Public Health. 2012;12:80. 10.1186/1471-2458-12-80. 22276600 10.1186/1471-2458-12-80 PMC3292515

[55] Hemming K, Taljaard M, Weijer C, Forbes AB. Use of multiple period, cluster randomised, crossover trial designs for comparative effectiveness research. BMJ. 2020;371:m3800. 10.1136/bmj.m3800. 33148538 10.1136/bmj.m3800

[56] Deutsche Rentenversicherung Nordbayern (DRV Nordbayern). Zahlen zu Nachsorgeleistungen der DRV Nordbayern, noch unveröffentlichter Geschäftsbericht 2024 [persönliche Mitteilung über E-Mail] [Figures on aftercare services of the DRV North Bavaria, as yet unpublished Annual Report 2024; personal communication via e-mail]. Bayreuth; 12 May 2025.

[57] Hemming K, Kasza J, Hooper R, Forbes A, Taljaard M. A tutorial on sample size calculation for multiple-period cluster randomized parallel, cross-over and stepped-wedge trials using the Shiny CRT Calculator. Int J Epidemiol. 2020;49(3):979–95. 10.1093/ije/dyz237. 32087011 10.1093/ije/dyz237 PMC7394950

[58] The Shiny CRT Calculator: power and sample size for cluster randomised trials. Accessed 17 July 2025. https://clusterrcts.shinyapps.io/rshinyapp/

[59] Leinberger S, Hetzel C, Kaluscha R. Adjustierung des sozialmedizinischen Verlaufs nach medizinischer Rehabilitation: methodische Weiterentwicklung der Reha-Qualitätssicherung der Deutschen Rentenversicherung [Risk adjustment of return to work after medical rehabilitation: Methodical advancements in quality assurance of the German Pension Insurance]. Rehabilitation. 2023;62(4):225–31. 10.1055/a-1998-6574. 36796424 10.1055/a-1998-6574

[60] Deck R, Hüppe A. Begleitete Nachsorge in der Psychosomatik - Transfer des neuen Credo [Supported aftercare into psychosomatic rehabilitation - transfer of new credo]. Rehabilitation. 2014;53(5):305–12. 10.1055/s-0034-1384594. 25188205 10.1055/s-0034-1384594

[61] Deck R, Schramm S, Hüppe A. Begleitete Eigeninitiative nach der Reha („neues Credo) - ein Erfolgsmodell? [Supported own initiative of rehabilitation patients (new credo) - a successful model?]. Rehabilitation. 2012;51(5):316–25. 10.1055/s-0031-1291279. 22473476 10.1055/s-0031-1291279

[62] Deck R, Beitz S, Baumbach C, Brunner S, Hoberg E, Knoglinger E. Nachsorge ‚Neues Credo‘ in der kardiologischen Anschlussrehabilitation [Rehabilitation aftercare ‘New Credo’ in the cardiac follow-up rehabilitation]. Rehabilitation. 2020;59(1):17–25. 10.1055/a-0899-1444. 31207652 10.1055/a-0899-1444

[63] Ernst G, Hübner P. Intervallrehabilitation bei Diabetes mellitus: Ergebnisse einer randomisierten kontrollierten Studie zur Nachsorge in der medizinischen Rehabilitation [Fractionated inpatient rehabilitation of diabetes: results from a randomized controlled trial on rehabilitation aftercare]. Rehabilitation. 2012;51(5):308–15. 10.1055/s-0031-1291282. 22477640 10.1055/s-0031-1291282

[64] Eusterbrock ST, Jochheim RJ, Badke M, Deck R. Effekte einer begleiteten Nachsorge in der Post-Reha-Phase bei COPD-Patienten: eine kontrollierte Studie [Effects of supported aftercare in the post-rehabilitation phase in COPD patients: A controlled study]. Pneumologie. 2021;75(12):929–41. 10.1055/a-1507-9057. 34171928 10.1055/a-1507-9057

[65] Huber D, Hoerschelmann N, Hoberg E, Karoff J, Karoff M, Kittel J. Berufsbezogene Rehabilitation und Nachsorge in der kardiologischen Rehabilitation (BERUNA): Ergebnisse einer randomisierten Kontrollgruppenstudie [Vocational inpatient and post-treatment proposals in cardiac rehabilitation patients (BERUNA): results of a randomized controlled trial]. Rehabilitation. 2014;53(6):362–8. 10.1055/s-0034-1384597. 25494343 10.1055/s-0034-1384597

[66] Prehn J, Remus L, Bethge M. Akzeptanz einer digitalen Rehabilitationsnachsorge bei Muskel-Skelett-Erkrankungen: eine Kohortenstudie [Acceptance of digital rehabilitation aftercare for musculoskeletal disorders: A cohort study]. Phys Rehab Kur Med. 2024. 10.1055/a-2439-4038.

[67] Schramm S, Hüppe A, Jürgensen M, Deck R. Begleitete Eigeninitiative nach der Reha („Neues Credo) - Langzeitergebnisse der quasiexperimentellen Interventionsstudie [Supported own initiative of rehabilitation patients (new credo) - longterm effects of the nonrandomized trial]. Rehabilitation. 2014;53(5):297–304. 10.1055/s-0033-1358388. 24399285 10.1055/s-0033-1358388

[68] Vogel M, Walther AL, Deck R. Telefonische sozialdienstliche Nachsorge zur Verbesserung der beruflichen Reintegration nach stationärer medizinischer Rehabilitation [Telephone aftercare by social services to improve return to work after medical rehabilitation]. Rehabilitation. 2017;56(6):379–88. 10.1055/s-0043-111614. 28759903 10.1055/s-0043-111614

[69] Becker J, Kreis A, Beutel ME, Zwerenz R. Wirksamkeit der internetbasierten, berufsbezogenen Nachsorge GSA-Online im Anschluss an die stationäre psychosomatische Rehabilitation: Ergebnisse einer randomisiert kontrollierten Studie [Effectiveness of the internet-based, job-related aftercare GSA-Online following inpatient psychosomatic rehabilitation: Results of a randomized controlled trial]. Rehabilitation. 2022;61(4):276–86. 10.1055/a-1871-4484. 35995057 10.1055/a-1871-4484

[70] Hass HG, Muthny F, Stepien J, Lerch J, Marwitz C, von der, Schröck R, et al. Effekte der telefonischen Nachsorge in der onkologischen Rehabilitation nach Brustkrebs – Ergebnisse einer randomisierten Studie [Effects of a phone-based follow-up care after inpatient rehabilitation for breast cancer patients - a randomized controlled trial]. Rehabilitation. 2017;56(3):189–97. 10.1055/s-0042-121384. 28599338 10.1055/s-0042-121384

[71] Meng K, Musekamp G, Schuler M, Seekatz B, Glatz J, Karger G, et al. The impact of a self-management patient education program for patients with chronic heart failure undergoing inpatient cardiac rehabilitation. Patient Educ Couns. 2016;99(7):1190–7. 10.1016/j.pec.2016.02.010. 26898600 10.1016/j.pec.2016.02.010

[72] Meng K, Reusch A, Musekamp G, Seekatz B, Zietz B, Steimann G, et al. Self-management education for rehabilitation inpatients: A cluster-randomized controlled trial. Patient Educ Couns. 2018;101(9):1630–8. 10.1016/j.pec.2018.03.027. 29627267 10.1016/j.pec.2018.03.027

[73] Meng K, Seekatz B, Haug G, Mosler G, Schwaab B, Worringen U, et al. Evaluation of a standardized patient education program for inpatient cardiac rehabilitation: impact on illness knowledge and self-management behaviors up to 1 year. Health Educ Res. 2014;29(2):235–46. 10.1093/her/cyt107. 24399262 10.1093/her/cyt107

[74] Peters M, Budde A, Jeising A, Lindner J, Schulz H. Behandlungsergebnisse in der psychosomatischen Rehabilitation – Die Hersfelder Katamnesestudie [Treatment outcomes in psychosomatic rehabilitation –. Hersfeld Catamnesis Study] Rehabilitation. 2022;61(4):240–9. . 10.1055/a-1865-1256 10.1055/a-1865-1256 35995054

[75] Salzwedel A, Heidler M-D, Meng K, Schikora M, Wegscheider K, Reibis R, et al. Impact of cognitive performance on disease-related knowledge six months after multi-component rehabilitation in patients after an acute cardiac event. Eur J Prev Cardiol. 2019;26(1):46–55. 10.1177/2047487318791609. 30073848 10.1177/2047487318791609

[76] Meng K, Heß V, Schulte T, Faller H, Schuler M. Health literacy bei onkologischen Rehabilitanden und deren Relevanz für den subjektiven Rehabilitationsverlauf [The impact of health literacy on health outcomes in cancer patients attending inpatient rehabilitation]. Rehabilitation. 2021;60(2):102–9. 10.1055/a-1361-4072. 33858019 10.1055/a-1361-4072

[77] Malterud K, Siersma VD, Guassora AD. Sample size in qualitative interview studies: Guided by information power. Qual Health Res. 2016;26(13):1753–60. 10.1177/1049732315617444. 26613970 10.1177/1049732315617444

[78] Kassenärztliche Vereinigung Bayerns (KVB). Versorgungsatlas Hausärzte. Darstellung der regionalen Versorgungssituation sowie der Altersstruktur in Bayern (Stand. Januar 2025) [General Practice Care Atlas. A presentation of the regional care situation and age structure in Bavaria (as of January 2025).]. 2025. https://www.kvb.de/fileadmin/kvb/Ueber-uns/Versorgungssituation/Versorgungsatlas/KVB-Versorgungsatlas-Hausaerzte.pdf. Accessed 5 November, 2025.

[79] Deck R, Träder J-M, Raspe H. Identifikation von potenziellem Reha-Bedarf in der Hausarztpraxis: Idee und Wirklichkeit [Identification of potential need for medical rehabilitation by general practitioners: idea and reality]. Rehabilitation. 2009;48(2):73–83. 10.1055/s-0028-1102952. 19421938 10.1055/s-0028-1102952

[80] Scholl I, Zill JM, Härter M, Dirmaier J. An integrative model of patient-centeredness - a systematic review and concept analysis. PLoS ONE. 2014;9(9):e107828. 10.1371/journal.pone.0107828. 25229640 10.1371/journal.pone.0107828 PMC4168256

