# Supplementary material for: Evaluation of a general practitioner-based rehabilitation follow-up consultation to promote patients’ use of medical rehabilitation aftercare: study protocol for a pragmatic cluster randomized crossover trial
Source: BMC Health Serv Res. 2026 Jun 12;26:815. doi: 10.1186/s12913-026-14938-9 (PMC13263927; doi:10.1186/s12913-026-14938-9)
Supplement: Supplementary file 2 — Additional file 2: WHO Trial Registration Data Set [file 12913_2026_14938_MOESM2_ESM.docx]

**WHO Trial Registration Data Set (Version 1.3.1)**

**“Evaluation of a general practitioner-based rehabilitation follow-up consultation to promote the use of aftercare services by rehabilitation patients: Study protocol for a pragmatic cluster randomized crossover trial”**

| *Item* | *Information* |
| --- | --- |
| **Primary Registry and Trial Identifying Number** | Deutsches Register Klinischer Studien DRKS (German Clinical Trials Register), ID DRKS00038353 |
| **Date of Registration in Primary Registry** | November 5, 2025 |
| **Secondary Identifying Numbers** | Reference number: Abteilung 31/4120 (§31/1/1/3 SGB VI - 38) |
| **Source(s) of Monetary or Material Support** | Deutsche Rentenversicherung Nordbayern (German Pension Insurance North Bavaria) |
| **Primary Sponsor** | Deutsche Rentenversicherung Nordbayern (German Pension Insurance North Bavaria) |
| **Secondary Sponsor(s)** | --- |
| **Contact for Public Queries** | Dr Matthias Lukasczik, Rehabilitation Sciences Working Group, Würzburg University Hospital, Würzburg, Germany; Lukasczik_M@ukw.de  Dr Jennifer Seeger, Rehabilitation Sciences Working Group, Würzburg University Hospital, Würzburg, Germany; Seeger_S@ukw.de |
| **Contact for Scientific Queries** | Dr Matthias Lukasczik, Rehabilitation Sciences Working Group, Würzburg University Hospital, Würzburg, Germany; Lukasczik_M@ukw.de  Dr Jennifer Seeger, Rehabilitation Sciences Working Group, Würzburg University Hospital, Würzburg, Germany; Seeger_S@ukw.de |
| **Public Title** | Rehabilitation Follow-Up Consultation (in German: Reha-Nachsorge-Gespräch) |
| **Scientific Title** | Evaluation of a general practitioner-based rehabilitation follow-up consultation to promote the use of aftercare services by rehabilitation patients (in German: Einbindung von niedergelassenen Ärztinnen und Ärzten in die Nachsorge nach medizinischer Rehabilitation; Acronym: RENAGE – Reha-Nachsorge-Gespräch) |
| **Countries of Recruitment** | Germany |
| **Health Condition(s) or Problem(s) Studied** | Medical rehabilitation (patients of all indications except oncology, psychosomatics, addictive disorders, child/adolescent rehabilitation) |
| **Intervention(s)** | Structured general practitioner-based follow-up consultation for patients following inpatient medical rehabilitation  Intervention group: usual recommendations for rehabilitation aftercare during rehabilitation plus invitation for follow-up consultation with GP  Control group: usual recommendations for rehabilitation aftercare during rehabilitation, no follow-up consultation with GP |
| **Key Inclusion and Exclusion Criteria** | Inclusion criteria – patients:  Adult medical rehabilitation patients aged 18 to 60 years insured with the German Pension Insurance North Bavaria who undergo a medical rehabilitation program in one of six cooperating inpatient rehabilitation centers during the recruitment period  Exclusion criteria – patients:  Age under 18 or over 60 years, AHB procedure (“Anschlussheilbehandlung”; medical rehabilitation immediately after hospitalization, e.g., following myocardial infarction or surgery), severe cognitive impairment, lack of understanding of the German language  Inclusion criteria – general practitioners:  GPs (including family doctors specializing in internal medicine) in the northern part of the federal state of Bavaria whose patients are undergoing medical rehabilitation at one of the cooperating rehabilitation centers and have agreed to participate in the study |
| **Study Type** | Interventional  Allocation: cluster randomized, crossover design, single-masked (patients)  Purpose: evaluation (can the intervention contribute to an increased utilization of rehabilitation aftercare following medical rehabilitation?) |
| **Date of First Enrollment** | Anticipated date: November 2025 |
| **Sample Size** | N = 348 medical rehabilitation patients  N = 77 general practitioners |
| **Recruitment Status** | Pending |
| **Primary Outcome(s)** | Self-reported utilization of rehabilitation aftercare 4 months post-rehabilitation |
| **Key Secondary Outcomes** | Self-reported work ability 4 months post-rehabilitation |
| **Ethics Review** | Status: approved  Date of approval: November 3, 2025  Ethics committee: Ethics committee of the University of Würzburg, Petrinistraße 33A, D-97080 Würzburg, Germany; E-Mail: ethikkommission@uni-wuerzburg.de |
| **Completion date** | Anticipated date: June 2027 |
| **Summary Results** | --- |
| **IPD sharing statement** | Sharing of deidentified individual clinical trial participant-level data (IPD) not planned |
