# Supplementary material for: Evaluation of a general practitioner-based rehabilitation follow-up consultation to promote patients’ use of medical rehabilitation aftercare: study protocol for a pragmatic cluster randomized crossover trial
Source: BMC Health Serv Res. 2026 Jun 12;26:815. doi: 10.1186/s12913-026-14938-9 (PMC13263927; doi:10.1186/s12913-026-14938-9)
Supplement: Supplementary file 3 — Additional file 3: SPIRIT flow diagram [file 12913_2026_14938_MOESM3_ESM.docx]

Additional file 3: Schedule of enrolment, interventions and assessments (cf. item 13 of the SPIRIT reporting checklist)

|  | STUDY PERIOD | | | | | |
| --- | --- | --- | --- | --- | --- | --- |
|  | Allocation (cluster randomization of MR facilities) | Enrolment | Post-allocation | | | Close-out |
| TIMEPOINT | - 1 | 0 | T1 | T2 | T3 | After 19 weeks post-enrolment |
|  |  | Start of MR | Completion of MR (3 weeks after enrolment) | GP follow-up consultation, 4-8 weeks after completion of MR (7-11 weeks after enrolment) | 4 months after completion of MR (19 weeks after enrolment) |  |
| ENROLMENT: |  | | | | | |
| Eligibility screen |  | **X** |  |  |  |  |
| Informed consent |  | **X** |  |  |  |  |
| Allocation | **X** |  |  |  |  |  |
| INTERVENTIONS: |  | | | | | |
| Intervention group (GP follow-up consultation) |  |  | **X** | **X** | **X** |  |
| Control group (care-as-usual) |  |  | **X** | **X** | **X** |  |
| ASSESSMENTS: |  | | | | | |
| Rehabilitation aftercare motivation |  |  | **X** | **X** | **X** |  |
| Rehabilitation aftercare self-efficacy |  |  | **X** | **X** | **X** |  |
| Rehabilitation aftercare recommendations by MR facility |  |  | **X** |  |  |  |
| Evaluation of medical rehabilitation outcomes |  |  | **X** | **X** | **X** |  |
| Self-reported work ability (WAS) |  |  | **X** | **X** | **X** |  |
| Health-related quality of life (EQ-5D 5L) |  |  | **X** | **X** | **X** |  |
| Health literacy (HLS19-Q12-DE) |  |  | **X** | **X** | **X** |  |
| Communicative self-efficacy in patient-physician interactions |  |  | **X** |  |  |  |
| Sociodemographic data |  |  | **X** |  |  |  |
| Use of/ participation in follow-up consultation (IG only) |  |  |  | **X** |  |  |
| Evaluation of follow-up consultation (IG only) |  |  |  | **X** |  |  |
| Implementation of aftercare recommendations by MR facility |  |  |  | **X** |  |  |
| Socio-medical data (return to work status) |  |  |  | **X** | **X** |  |
| Rehabilitation aftercare initiation/ continuation (primary outcome) |  |  |  |  | **X** |  |
